# Neuroprotective actions of cerebellar and pineal allopregnanolone on Purkinje cells

**DOI:** 10.1096/fba.2019-00055

**Published:** 2020-02-05

**Authors:** Kazuyoshi Tsutsui, Shogo Haraguchi

**Affiliations:** ^1^ Laboratory of Integrative Brain Sciences Department of Biology Waseda University Center for Medical Life Science of Waseda University Tokyo Japan; ^2^ Department of Biochemistry Showa University School of Medicine Tokyo Japan

**Keywords:** allopregnanolone, brain and pineal gland, neuronal survival, progesterone, Purkinje cell

## Abstract

The brain produces steroids de novo from cholesterol, so‐called “neurosteroids.” The Purkinje cell, a cerebellar neuron, was discovered as a major site of the biosynthesis of neurosteroids including sex steroids, such as progesterone, from cholesterol in the brain. Allopregnanolone, a progesterone metabolite, is also synthesized in the cerebellum and acts on the Purkinje cell to prevent cell death of this neuron. Recently, the pineal gland was discovered as an important site of the biosynthesis of neurosteroids. Allopregnanolone, a major pineal neurosteroid, acts on the Purkinje cell for the survival of this neuron by suppressing the expression of caspase‐3, a crucial mediator of apoptosis. This review summarizes the discovery of cerebellar and pineal allopregnanolone and its neuroprotective action on Purkinje cells.

**Abbreviations**
3α‐HSD: 3α‐hydroxysteroid dehydrogenase
3β‐HSD: 3β‐hydroxysteroid dehydrogenase/Δ^5^‐Δ^4^‐isomerase
17β‐HSD: 17β‐hydroxysteroid dehydrogenase
BDNF: brain‐derived neurotrophic factor
ER: endoplasmic reticulum
ERE: estrogen response element
ERs: estrogen receptors
GABA_A_: γ‐aminobutyric acid type A
GC‐MS: gas chromatography‐mass spectrometry
Golgi: Golgi apparatus
HPLC: high‐performance liquid chromatography
IGF‐1: insulin‐like growth factor‐1
NP‐C: Niemann‐Pick type C
NT‐3: neurotrophin‐3
P450_17α,lyase_: cytochrome P450 17α‐hydroxylase/c17,20‐lyase
P450arom: cytochrome P450 aromatase
P450scc: cytochrome P450 side‐chain cleavage enzyme
PACAP: pituitary adenylate cyclase‐activating polypeptide
PGRMC1: progesterone receptor membrane component 1
PRE: progesterone response element
PRs: progesterone receptors
RT‐PCR: reverse transcription‐polymerase chain reaction
RU486: mifepristone
StAR: steroidogenic acute regulatory protein
TrkB: BDNF receptor

## 1 INTRODUCTION

It was traditionally believed that the brain is a target site for peripheral steroid hormones that act on the brain to regulate several important brain functions. Moreover, extensive studies over the past 30 years have demonstrated that the brain also produces steroids de novo from cholesterol, so‐called “neurosteroids” (for reviews, see 1, 2). The formation of neurosteroids in the brain was originally discovered in mammals and subsequently in other vertebrates. Now it is accepted that the biosynthesis of neurosteroids from cholesterol in the brain is highly conserved across vertebrate species (for reviews, see 1, 2).

To investigate the biological actions of neurosteroids in the brain, the identification of neurosteroidogenic cells is essential. Glial cells, such as oligodendrocytes and astrocytes, were first identified as the primary site for the biosynthesis of neurosteroids in the brain of mammals and other vertebrates (for reviews, see 1, 3). Moreover, biosynthesis of neurosteroids in the brain neuron was unknown for a while in any vertebrate.

From the extensive studies, however, the Purkinje cell, an important cerebellar neuron, has been discovered as a major neurosteroidogenic cell in the brain of various vertebrates.^10^, ^11^, ^12^, ^13^, ^14^, ^15^, ^16^, ^17^ The Purkinje cell is known to play important roles as a cerebellar neuron in the process of memory and learning and other cerebellar functions.^18^ The discovery of the biosynthesis of neurosteroids in the Purkinje cell gave a great impact on neurosteroid research. Importantly, the Purkinje cell expresses key steroidogenic enzymes, cytochrome P450 side‐chain cleavage enzyme (P450scc), and 3β‐hydroxysteroid dehydrogenase/∆^5^‐∆^4^‐isomerase (3β‐HSD), and actively produces progesterone de novo from cholesterol during cerebellar development.^12^, ^13^, ^19^ The biosynthesis and biological action of progesterone have been reported.^20^, ^21^, ^22^, ^23^, ^24^, ^25^, ^26^ Biochemical studies further showed that the Purkinje cell produces allopregnanolone (3α,5α‐tetrahydroprogesterone), a progesterone metabolite, by the steroidogenic enzymes, 5α‐reductase and 3α‐HSD, during cerebellar development.^27^, ^28^, ^29^, ^30^ Subsequent studies using the Purkinje cell have demonstrated the neurotrophic action of progesterone on the growth of Purkinje cells^23^, ^24^, ^25^, ^26^ and the neuroprotective action of allopregnanolone on the survival of Purkinje cells during cerebellar development.^31^ Thus, these studies taken together have demonstrated the biosynthesis and biological actions of neurosteroids produced in the Purkinje cell in mammals and other vertebrates (for reviews, see 4, 5, 6).

Subsequently, the biosynthesis of neurosteroids de novo from cholesterol in the pineal gland, an endocrine organ located close to the cerebellum, was discovered.^32^, ^33^ Although for the past 30 years, it was generally believed that only neurons and glial cells in the brain produce neurosteroids (for reviews, see 1, 3, 4), thus the discovery of pineal neurosteroids has built a new concept of the biosynthesis of neurosteroids. Importantly, allopregnanolone is produced as a major pineal neurosteroid and secreted from the pineal gland during cerebellar development.^32^, ^33^ The follow‐up studies have further demonstrated the neuroprotective action of pineal allopregnanolone that is involved in the survival of Purkinje cells by suppressing the activity of caspase‐3, a crucial mediator of apoptosis during cerebellar development^32^ (for a review, see 34).

This review describes the advances made in our understanding of neuroprotective actions of cerebellar and pineal allopregnanolone on Purkinje cells during cerebellar development.

## 2 IDENTIFICATION OF THE PURKINJE CELL AS A MAJOR SITE OF THE BIOSYNTHESIS OF NEUROSTEROIDS

**Figure 1:**
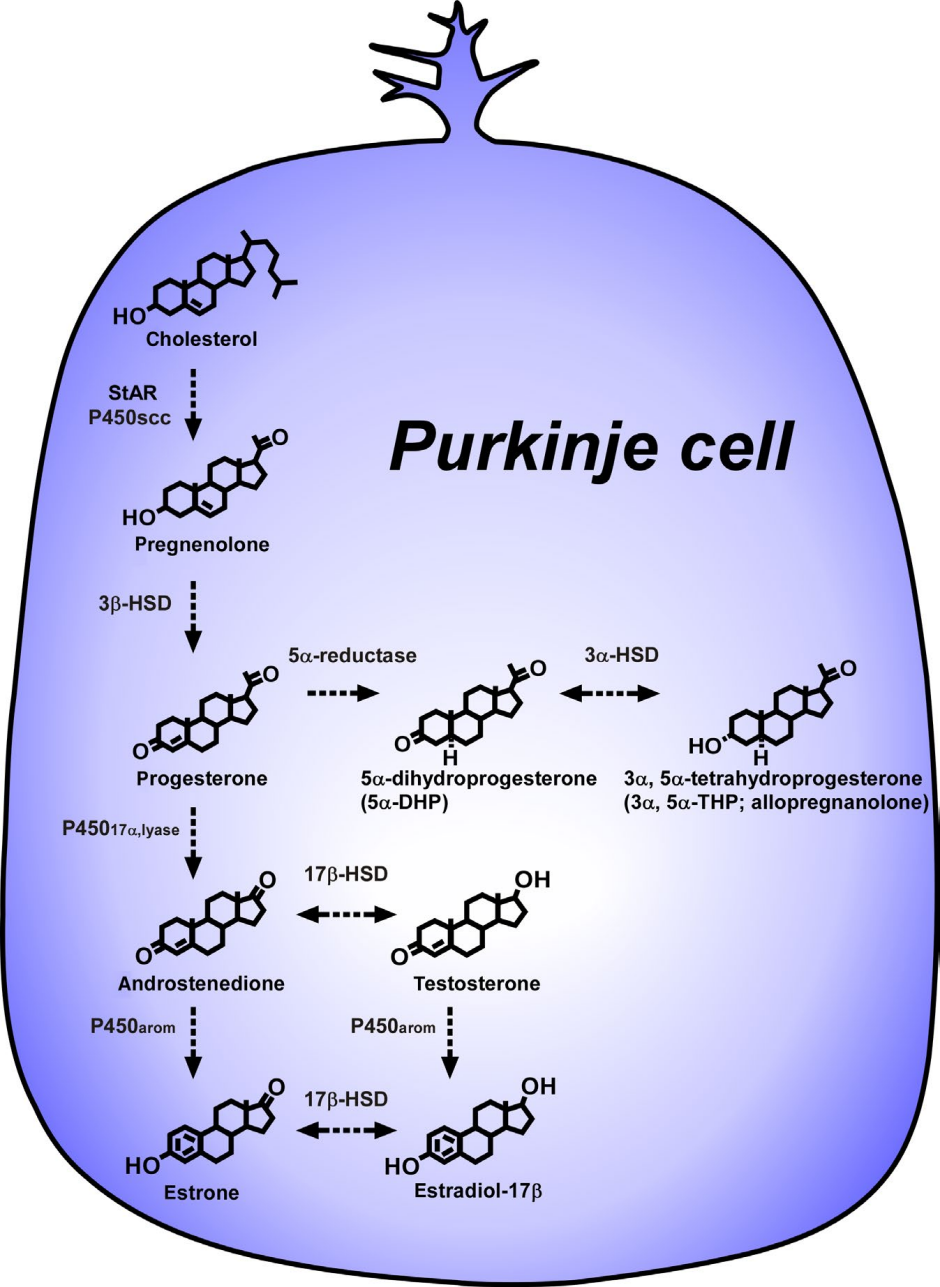
Biosynthesis of neurosteroids in the cerebellar Purkinje cell. The Purkinje cell, an important cerebellar neuron, is a major site for the biosynthesis of neurosteroids in the brain. The Purkinje cell expresses steroidogenic enzymes that produce a variety of neurosteroids. The Purkinje cell produces progesterone from pregnenolone actively due to an increase in 3β‐HSD activity during cerebellar development. Allopregnanolone (3α,5α‐tetrahydroprogesterone) is also metabolized by the enzymes 5α‐reductase and 3α‐HSD from progesterone during neonatal development. The Purkinje cell further expresses P45017α,lyase and P450arom, and actively produces estradiol‐17β during neonatal development. 17β‐HSD, 17β‐hydroxysteroid dehydrogenase; 3α‐HSD, 3α‐hydroxysteroid dehydrogenase; 3β‐HSD, 3β ‐hydroxysteroid dehydrogenase/Δ^5^‐Δ^4^‐isomerase; P450_17α,lyase_, cytochrome P450 17α‐hydroxylase/c17,20‐lyase; P450arom, cytochrome P450 aromatase; P450scc, cytochrome P450 side‐chain cleavage enzyme; StAR, steroidogenic acute regulatory protein

Many groups worldwide have dedicated their researches to understand various crucial roles in the cerebellum (for a review, see 18). These studied taken together have established that the Purkinje cell, an important cerebellar neuron, contributes to several cerebellar functions (for a review, see 18). Importantly, de novo neuronal biosynthesis of neurosteroids from cholesterol has been demonstrated in the Purkinje cell by immunohistochemical and biochemical studies.^23^, ^24^, ^25^, ^26^ Pregnenolone, a 3β‐hydroxy‐Δ^5^‐steroid, is the main precursor of steroid hormones and the biosynthesis of pregnenolone is initiated by the cleavage of the cholesterol side‐chain by cytochrome P450scc, a rate‐limiting mitochondrial enzyme. The expression of cytochrome P450scc in the Purkinje cell in quail was found by immunohistochemical and biochemical studies.^10^, ^11^ This was the first evidence for the neuronal location of cytochrome P450scc and the biosynthesis of pregnenolone in the brain neuron. To put avian findings into a broader perspective, the expression of cytochrome P450scc in the Purkinje cell of mammals was further demonstrated^12^ (Figure 1). Cytochrome P450scc is expressed in the Purkinje cell after its differentiation and the expression of this enzyme persists until adulthood in rats.^12^ In addition to birds and mammals, the expression of cytochrome P450scc in the Purkinje cell was further found in amphibians and fish.^15^, ^17^ It thus appears that the Purkinje cell expresses cytochrome P450scc and produces pregnenolone de novo from cholesterol across vertebrates (Figure 1). The Purkinje cell also expresses steroidogenic acute regulatory protein (StAR)^19^ (Figure 1) that acts on the transport of cholesterol to the inner mitochondrial membrane that localizes cytochrome P450scc.^35^

## 3 BIOSYNTHESIS OF PROGESTERONE AND ALLOPREGNANOLONE IN PURKINJE CELLS

After the discovery of pregnenolone biosynthesis in the Purkinje cell, subsequent studies have demonstrated that the Purkinje cell is a major site of the biosynthesis of various neurosteroids, such as progesterone and allopregnanolone, a progesterone metabolite, in the brain of vertebrates including mammals^12^, ^13^, ^17^, ^26^, ^27^, ^28^, ^29^, ^30^ (Figure 1). The colocalization of key steroidogenic enzymes in the Purkinje cell during development has been demonstrated (Figure 1). Subsequently, it was found de novo biosynthesis of progesterone by 3β‐HSD, a membrane‐bound mitochondrial enzyme from cholesterol in the Purkinje cell of rats during neonatal development^13^ (Figure 1). Now it is widely accepted that the Purkinje cell expresses both cytochrome P450scc and 3β‐HSD in rats (Figure 1). The expression of 3β‐HSD in the Purkinje cell is also evident in other vertebrates.^17^ The expression of 3β‐HSD and biosynthesis of progesterone in Purkinje cells increase during neonatal development in rats.^23^, ^24^, ^26^, ^27^, ^28^, ^29^, ^30^ Based on these studies, it has been demonstrated that the Purkinje cell actively produces progesterone during neonatal development^13^ (Figure 1).

Importantly, the biosynthesis of allopregnanolone (3α,5α‐tetrahydroprogesterone) in the cerebellum was further demonstrated by biochemical studies combined with high‐performance liquid chromatography (HPLC) and gas chromatography‐mass spectrometry (GC‐MS) analyses^5^, ^27^, ^28^, ^30^ (Figure 1). Biochemical and immunohistochemical studies showed that the Purkinje cell metabolizes progesterone to allopregnanolone by 5α‐reductase and 3α‐HSD during neonatal development (Figure 1). These studies taken together establish that the Purkinje cell expresses cytochrome P450scc, 3β‐HSD, 5α‐reductase and 3α‐HSD, and produces not only progesterone but also allopregnanolone de novo from cholesterol during neonatal development (Figure 1).

## 4 NEUROPROTECTIVE ACTION OF ALLOPREGNANOLONE PRODUCED IN PURKINJE CELLS

The Purkinje cell has served as an excellent cellular model for the study of biological actions of neurosteroids, because this neuron is a major site for the biosynthesis of neurosteroids in the brain. As mentioned above, the Purkinje cell actively synthesizes progesterone de novo from cholesterol during neonatal development in neonatal rats^13^ (Figure 1). The Purkinje cell also produces allopregnanolone, a progesterone metabolite, in neonatal rats.^27^, ^28^, ^29^, ^30^ It is well established in rats that marked morphological changes occur in the cerebellum during neonatal development.^36^, ^37^ The Purkinje cell differentiates around birth and the formation of the cerebellar cortex becomes complete during neonatal development through the processes of neuronal and glial growth, synaptogenesis and migration of external granule cells in rats.^36^, ^37^ Thus, cerebellar development is remarkable during neonatal life, when the biosynthesis of progesterone and allopregnanolone increases in the Purkinje cell of neonatal rats.^13^, ^27^, ^28^ Based on these morphological and biochemical observations, it is considered that progesterone and/or allopregnanolone may play crucial roles in the formation of cerebellar neuronal circuits by the promotion of neuronal growth and neuronal synaptic contact in the cerebellum during neonatal development.

Therefore, the biological actions of progesterone and allopregnanolone produced as major neurosteroids in the Purkinje cell on neuronal growth, spinogenesis, and synaptogenesis in the developing cerebellum were investigated during neonatal development. In vitro and in vivo studies using neonatal rats showed that progesterone promotes dendritic growth, dendritic spine, and dendritic spine synapse formation of the Purkinje cell.^23^, ^24^ In contrast to progesterone, allopregnanolone did not affect the growth of Purkinje cells during neonatal development.^23^, ^24^ Consequently, it is considered that progesterone was synthesized in the Purkinje cell acts on the promotion of the dendritic growth, spinogenesis, and synaptogenesis of Purkinje cells during cerebellar development. Moreover, it has been reported that the neuroprotection of Purkinje cells from developmental cell death by the anti‐progesterone RU486 suggests a role of progesterone in the protection of Purkinje cells.^25^ The possible actions of progesterone through membrane progesterone receptors (mPRs, PAQR family^22^) and microtubule modulation to produce neurotrophic and neuroprotective effects^20^, ^21^ have been reported.

As mentioned above, the Purkinje cell metabolizes progesterone to allopregnanolone during cerebellar development.^27^, ^28^, ^29^, ^30^ It has been reported that allopregnanolone is involved in the survival of Purkinje cells and granule cells,^31^ although allopregnanolone did not promote the dendritic growth, spinogenesis, and synaptogenesis of Purkinje cells during cerebellar development.^23^, ^24^ The Niemann‐Pick type C (NP‐C) mouse has been used as an excellent animal model^31^ for the investigation of the neuroprotective action of allopregnanolone. NP‐C is an autosomal recessive, childhood neurodegenerative disease characterized by defective intracellular cholesterol trafficking, resulting in the degeneration of Purkinje cells. It was found that the brain of NP‐C mice contains less allopregnanolone than that of wild‐type mice.^31^ In addition, the administration of allopregnanolone to neonatal NP‐C mice increases Purkinje cell survival and delays neurodegeneration.^31^ Thus, this study indicates that allopregnanolone is an important neurosteroid for Purkinje cell survival during cerebellar development. This is the first observation showing the neuroprotective action of allopregnanolone in the cerebellum.

## 5 MODE OF ACTION AND FUNCTIONAL SIGNIFICANCE OF NEUROSTEROIDS PRODUCED IN PURKINJE CELLS

To demonstrate the mode of action of progesterone produced in the Purkinje cell, the expression of progesterone receptors (PRs) in the cerebellum has been investigated during cerebellar development. It was found that the Purkinje cell expresses intranuclear PR‐A and PR‐B in neonatal rats.^23^, ^24^, ^26^ These studies indicate that progesterone can act directly on the Purkinje cell through intranuclear receptor‐mediated mechanisms to promote Purkinje dendritic growth, spinogenesis, and synaptogenesis during cerebellar development.^23^, ^24^, ^26^ Moreover, 25‐Dx, a putative membrane progesterone receptor, is also expressed in the Purkinje cell during cerebellar development.^38^ Therefore, it is considered that progesterone may promote dendritic growth, spinogenesis, and synaptogenesis *via* 25‐Dx as well as intranuclear PR‐A and PR‐B in the Purkinje cell during cerebellar development.^39^ 25‐Dx is now named “progesterone receptor membrane component 1” (PGRMC1). There are several studies showing that PGRMC1 mediates the anti‐apoptotic actions of progesterone that associate with plasminogen activator inhibitor RNA‐binding protein‐1 (PAIRBP1).^40^, ^41^, ^42^, ^43^

It has been reported that neurotrophic factors, such as brain‐derived neurotrophic factor (BDNF) and neurotrophin‐3 (NT‐3), are highly expressed in the cerebellum during development and are involved in the growth of Purkinje cells and granule cells.^44^, ^45^, ^46^, ^47^, ^48^ These previous studies indicate that BDNF and NT‐3 are attractive candidate regulators of Purkinje dendrite and spine development. Therefore, BDNF and/or NT‐3 may mediate the neurotrophic action of progesterone on Purkinje dendritic growth, spinogenesis, and synaptogenesis during cerebellar development (for reviews, see 49, 50).

## 6 IDENTIFICATION OF THE PINEAL GLAND AS A MAJOR SITE OF THE BIOSYNTHESIS OF NEUROSTEROIDS

**Figure 2:**
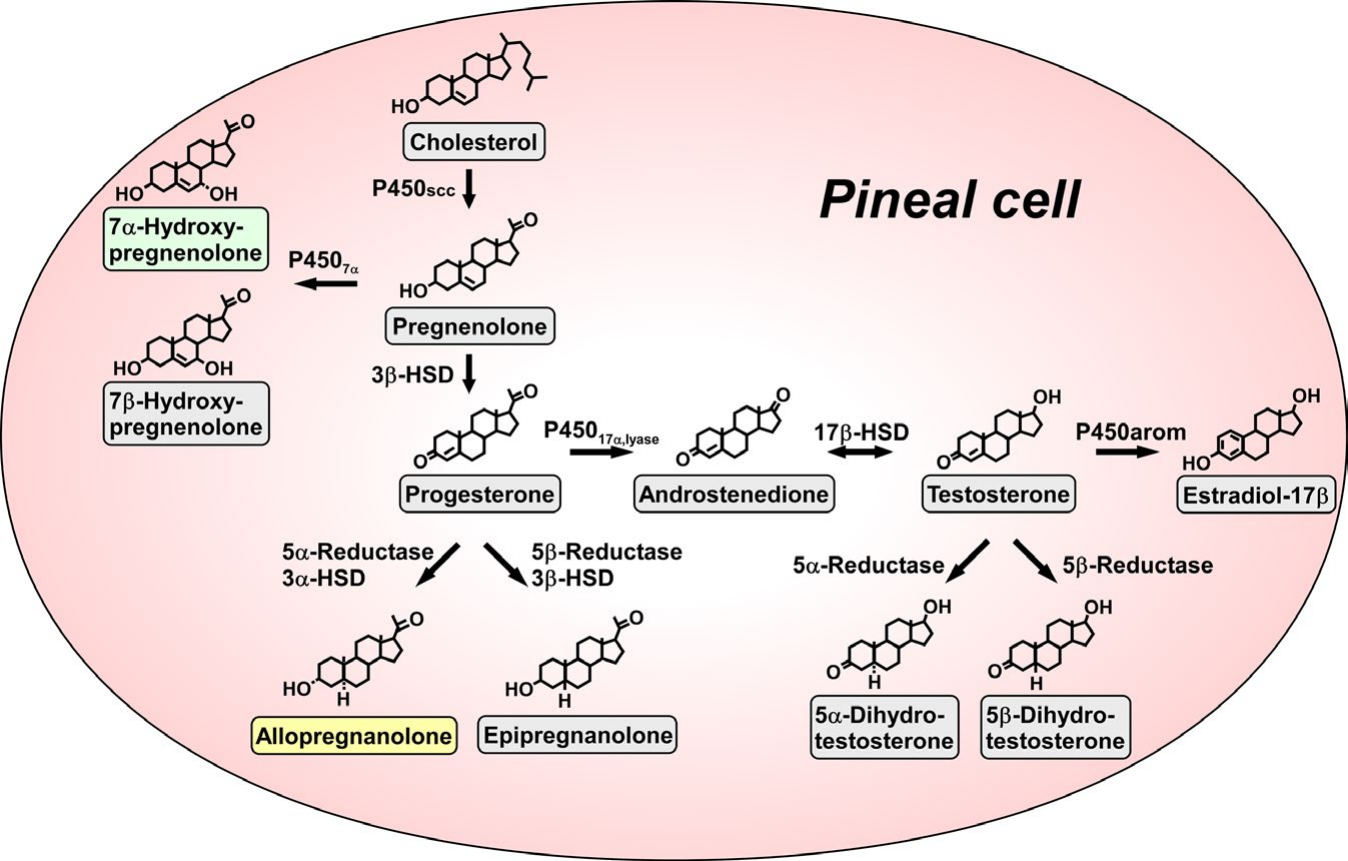
Biosynthesis of neurosteroids in the pineal gland. The pineal gland, an endocrine organ located close to the cerebellum, expresses several kinds of steroidogenic enzymes. The pineal gland produces a variety of neurosteroids, such as pregnenolone, 7α‐ and 7β‐hydroxypregnenolone (7α‐ and 7β‐OH pregnenolone), progesterone, allopregnanolone, androstenedione, testosterone, 5α‐ and 5β‐dihydrotestosterone, and estradiol‐17β. Allopregnanolone and 7α‐OH pregnenolone are the major pineal neurosteroids that are secreted from the pineal gland. 17β‐HSD, 17β‐hydroxysteroid dehydrogenase; 3α‐HSD, 3α‐hydroxysteroid dehydrogenase; 3β‐HSD, 3β‐hydroxysteroid dehydrogenase/Δ^5^‐Δ^4^‐isomerase; P450_17α,lyase_, cytochrome P450 17α‐hydroxylase/c17,20‐lyase; P450arom, cytochrome P450 aromatase; P450scc, cytochrome P450 side‐chain cleavage enzyme

Here, this review highlight the recent discovery of neurosteroids produced in the pineal gland. It is well known that the pineal gland is a photosensitive endocrine organ located close to the cerebellum and transduces photoperiodic changes in the neuroendocrine system by rhythmic melatonin secretion in vertebrates. Until recently, the biosynthesis of neurosteroids in the pineal gland remained unknown in any vertebrate. However, at the beginning of 2010s, the pineal gland was discovered as a major site for the biosynthesis of neurosteroids from cholesterol by molecular and biochemical studies^32^, ^33^ (Figure 2). For the past 30 years, it was generally accepted that neurosteroids are produced in neurons and glial cells which are located in the brain and peripheral nervous system (for reviews, see 1, 2). The discovery of neurosteroids produced in the pineal gland has built a new concept of the biosynthesis of neurosteroids.

As described above, pregnenolone is the common precursor of all steroid hormones and the formation of pregnenolone is initiated by cleavage of the side‐chain of cholesterol by cytochrome P450scc in vertebrates. It was first found that cytochrome P450scc is highly expressed in the pineal gland of juvenile chickens^33^ and juvenile quail^32^ by RT‐PCR analysis (Figure 2). The biosynthesis of pregnenolone from cholesterol in the pineal gland of these juvenile birds was also demonstrated by HPLC and GC‐MS analyses^32^, ^33^ (Figure 2). Subsequently, the localization of cytochrome P450scc in pinealocytes in the pineal gland was identified by immunohistochemical analysis^32^ (Figure 2). Molecular and biochemical analyses further showed that the pineal gland of juvenile chickens^33^ and juvenile quail^32^ expresses other key steroidogenic enzymes, such as cytochrome P450_7α_, 3α‐ and 3β‐HSD, 5α‐ and 5β‐reductase, cytochrome P450_17α,lyase_, 17β‐hydroxysteroid dehydrogenase (17β‐HSD), and cytochrome P450arom (Figure 2). Based on these new findings, the biosynthetic pathways of neurosteroids in the pineal gland have been demonstrated^32^, ^33^ as summarized in Figure 2. The pineal gland produces a variety of neurosteroids, such as pregnenolone, 7α‐, and 7β‐hydroxypregnenolone (7α‐ and 7β‐OH pregnenolone), progesterone, allopregnanolone, androstenedione, testosterone, 5α‐ and 5β‐dihydrotestosterone, and estradiol‐17β (Figure 2). These up‐to‐date studies provide the first evidence for de novo neurosteroidogenesis in the pineal gland in any vertebrate class.

## 7 IDENTIFICATION OF ALLOPREGNANOLONE AS A MAJOR PINEAL NEUROSTEROID

To understand the biological actions of neurosteroids produced in the pineal gland, the identification of major pineal neurosteroids is important. Biochemical studies combined with HPLC and GC‐MS analyses showed that allopregnanolone and 7α‐OH pregnenolone are major neurosteroids produced in the pineal gland^32^ (Figure 2). Pregnenolone is converted primarily into allopregnanolone and 7α‐OH pregnenolone in the pineal gland.^32^ The biosynthesis of these major pineal neurosteroids is higher in juvenile birds than in adult birds.^32^ Importantly, the biosynthesis of allopregnanolone and 7α‐OH pregnenolone is higher in the pineal gland than in the brain.^32^ Moreover, pineal allopregnanolone and 7α‐OH pregnenolone are released from the pineal gland.^32^, ^33^ Based on these new findings, allopregnanolone and 7α‐OH pregnenolone are identified as the major pineal neurosteroids that are secreted from the pineal gland of birds during development (Figure 2).

## 8 NEUROPROTECTIVE ACTION OF PINEAL ALLOPREGNANOLONE ON THE SURVIVAL OF PURKINJE CELLS

**Figure 3:**
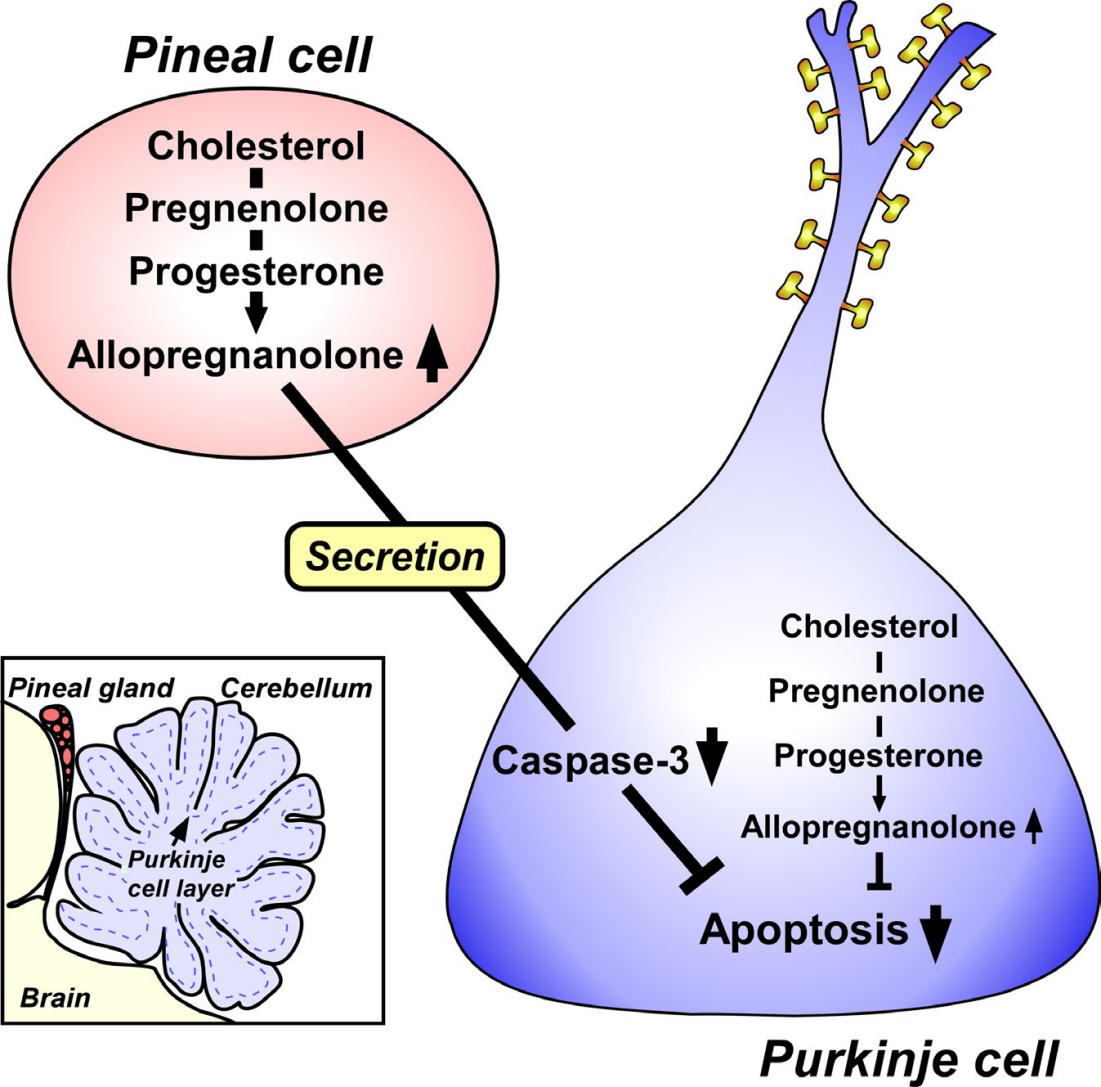
Neuroprotective action of allopregnanolone produced in the pineal gland on the survival of Purkinje cells during cerebellar development. Allopregnanolone that is actively produced in the pineal gland affects adjacent cerebellar Purkinje cells by diffusion and saves Purkinje cells from apoptosis in the developing cerebellum. Secreted pineal allopregnanolone inhibits the expression of active caspase‐3 that facilitates apoptosis of Purkinje cells during cerebellar development. Allopregnanolone is also synthesized in Purkinje cells and acts on the survival of this neuron

The two major pineal neurosteroids, allopregnanolone and 7α‐OH pregnenolone, may play crucial roles in the brain during development. Previous studies on birds and mammals indicate that pinealectomy (Px) induces cell loss in the brain including Purkinje cells during development.^51^, ^52^ Based on these findings, the biological action of allopregnanolone and 7α‐OH pregnenolone produced in the pineal gland on the survival of Purkinje cells during cerebellar development was investigated using juvenile birds. It was found that Px induces the decrease in allopregnanolone concentration in the cerebellum and the increase in apoptosis of Purkinje cells.^32^ It was further found that the administration of allopregnanolone to Px birds increases allopregnanolone concentration in the cerebellum and prevents apoptosis of Purkinje cells^32^ (Figure 3). Moreover, injection of ^3^H‐allopregnanolone close to the pineal lumen showed that pineal allopregnanolone reaches Purkinje cells in the cerebellum by diffusion (Figure 3).^32^ Consequently, these findings indicate that pineal allopregnanolone acts on Purkinje cell survival during cerebellar development (Figure 3). This is a new function of the pineal gland for the prevention of Purkinje cell death via pineal allopregnanolone during cerebellar development. In contrast to allopregnanolone, there was no effect of the other major pineal neurosteroid 7α‐OH pregnenolone on the survival of Purkinje cells.^32^ The discovery of the biosynthesis and neuroprotective action of pineal allopregnanolone on the survival of cerebellar Purkinje cells are new findings.

## 9 MODE OF ACTION OF AND FUNCTIONAL SIGNIFICANCE OF PINEAL ALLOPREGNANOLONE ON THE SURVIVAL OF PURKINJE CELLS

To understand the mode of action of pineal allopregnanolone on Purkinje cell survival, the factor that mediates the neuroprotective action of pineal allopregnanolone on Purkinje cells was subsequently investigated. Previous studies revealed that caspase‐3 plays an important role as a crucial mediator of apoptosis in Purkinje cell death in vertebrates.^53^, ^54^, ^55^ It was found that Px increases the number of Purkinje cells that express active caspase‐3 in juvenile birds and the administration of allopregnanolone to Px birds decreases the number of Purkinje cells expressing active caspase‐3.^32^ These findings indicate that the neuroprotective action of pineal allopregnanolone on Purkinje cells is accompanied by the decrease in caspase‐3 activity during cerebellar development. Accordingly, it is considered that allopregnanolone produced in the pineal gland exerts an antiapoptotic action on Purkinje cells in the cerebellum by suppressing caspase‐3 activity during cerebellar development (Figure 3).

**Figure 4:**
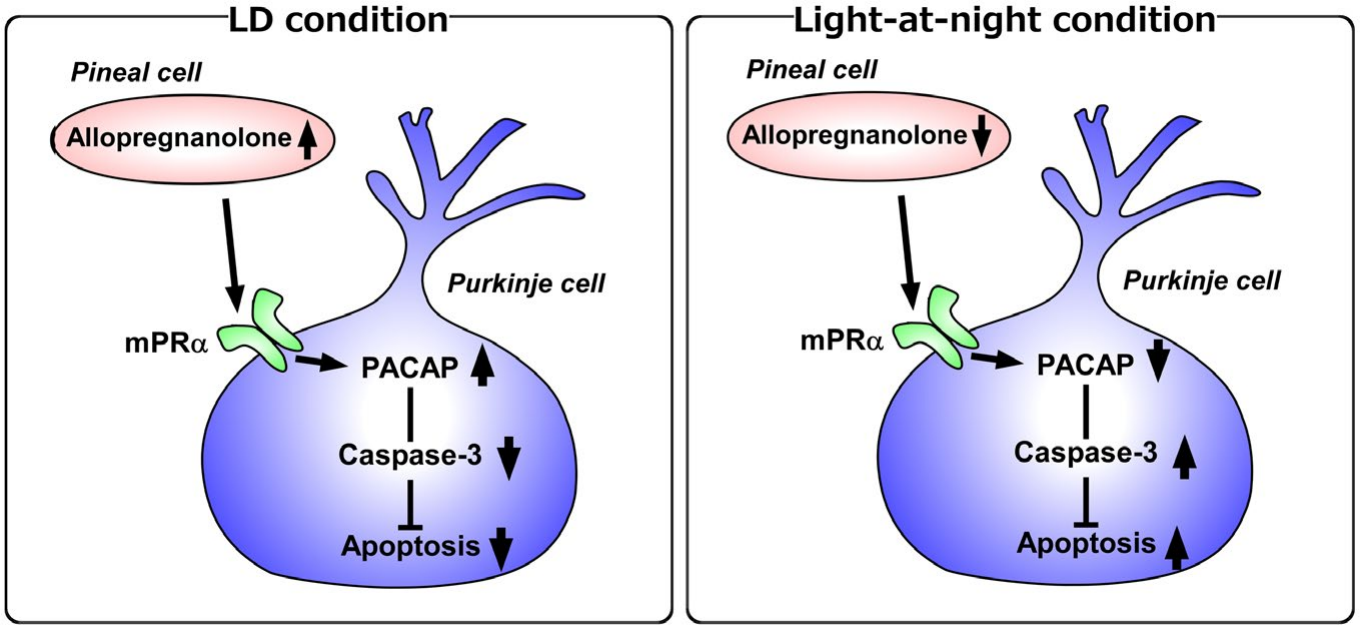
A schematic model of the effect of pineal allopregnanolone on Purkinje cell survival under LD or light‐at‐night conditions. (*Left panel*) A schematic model of normal development of the cerebellum under LD conditions. Pineal allopregnanolone induces the expression of PACAP through the mPRα mechanism in Purkinje cells. Then, PACAP inhibits the expression of active caspase‐3 that may facilitate the apoptosis of Purkinje cells in the cerebellum. (*Right panel*) A schematic model of the abnormal development of the cerebellum under light‐at‐night conditions. Light‐at‐night conditions disrupted the diurnal rhythm in pineal allopregnanolone synthesis. Decreased pineal allopregnanolone synthesis decreases the expression of PACAP in Purkinje cells. Following this, increased amounts of caspase‐3 facilitate the apoptosis of Purkinje cells in the cerebellum

Until now, it is unclear whether the action of pineal allopregnanolone on caspase‐3 activity in the Purkinje cell is mediated through a membrane receptor or involving transcriptional activation. Thus, the intracellular signaling pathway exerting a neuroprotective action of allopregnanolone produced in the brain remains unclear. Moreover, it is known that allopregnanolone is a potent allosteric modulator of the γ‐aminobutyric acid type A (GABA_A_) receptor in the brain.^56^, ^57^ There are reports that indicate the biological action of allopregnanolone produced in the brain is likely mediated through interaction with the pathway of the GABA_A_ receptor. To demonstrate the intracellular signaling pathway exerting a neuroprotective action of allopregnanolone in the Purkinje cell, characterization of the mode of action of pineal allopregnanolone on the suppression of caspase‐3 activity in the Purkinje cell during cerebellar development has been investigated. Allopregnanolone acts as an agonist of the GABA_A_ receptor and may act as an agonist of the mPRα, mPRβ, and mPRγ.^58^, ^59^, ^60^ To investigate the identified putative receptors that mediate the neuroprotective action of pineal allopregnanolone, either mPRs siRNAs or isoallopregnanolone, an antagonist of allopregnanolone, were delivered into the cerebellum of chicks. It was found that silencing of mPRα increases the number of Purkinje cells that expressed active caspase‐3 in the cerebellum of chicks relative to the number in the control siRNA^61^ (Figure 4). Furthermore, to clarify the neuroprotective mechanisms of allopregnanolone in Purkinje cells, the effect of allopregnanolone on the expression of neuroprotective and neurotoxic factors^62^, ^63^, ^64^, ^65^, ^66^, ^67^ was investigated. Px decreased the expression of pituitary adenylate cyclase‐activating polypeptide (PACAP), a neuroprotective factor, mRNA in the cerebellum of juvenile birds^61^ (Figure 4). It was found that a daily injection of allopregnanolone in Px juvenile birds increases the expression of PACAP relative to that in Px birds^61^ (Figure 4). Caspase‐3 is a key enzyme involved in the neuroprotective action of PACAP.^68^ Based on these new findings, PACAP mediates the neuroprotective action of pineal allopregnanolone through the mPRα mechanism during cerebellar development (Figure 4).

For the understanding of the functional significance of pineal allopregnanolone in the cerebellum, investigating the regulatory mechanisms of the biosynthesis of pineal allopregnanolone is important. It is known that environmental stimuli (eg, light‐dark cycle, temperature, or nutrition) influence the development of plants, animals, and humans. Especially, the light‐dark cycle strongly affects the development. Previous studies have demonstrated that a light‐dark cycle promotes better brain development than does constant light or constant darkness.^69^, ^70^, ^71^, ^72^, ^73^ However, little is known about the molecular mechanisms that control how environmental light conditions affect brain development. As mentioned above, the pineal gland, a photosensitive organ located close to the cerebellum, actively produces allopregnanolone during cerebellar development. To investigate whether light conditions are involved in pineal allopregnanolone synthesis in juvenile birds, the birds were incubated under either a 12 hours light‐12 hours dark (LD) cycle or a 12 hours light‐12 hours dark cycle followed by exposure to light for 1 hour during the dark period (light‐at‐night). It was found the allopregnanolone concentration and synthesis are higher during the dark period in the pineal glands of LD birds than during the dark period in the pineal glands of light‐at‐night birds^61^ (Figure 4). It was further found that Purkinje cell numbers are decreased in the cerebellum by light‐at‐night during^61^ (Figure 4). It is, therefore, considered that pineal allopregnanolone contributes as an important internal factor depending on the environmental light conditions to affect cerebellar development in vertebrates.

It is known nocturnal secretion of melatonin by the pineal gland in photoperiodic vertebrates.^74^ Additionally, there is an important finding, indicating that melatonin modifies neurosteroid milieu in the brain.^75^ It has been demonstrated that melatonin regulates the biosynthesis of 7α‐OH pregnenolone in the diencephalon of birds.^75^ Concomitant Px and orbital enucleation (Ex) provoke a marked increase in the biosynthesis of 7α‐OH pregnenolone and stimulate the expression of cytochrome P450_7α_‐producing 7α‐OH pregnenolone in the quail diencephalon.^75^ In contrast, melatonin administration to Px/Ex quail decreases the biosynthesis of 7α‐OH pregnenolone and inhibits the expression of cytochrome P450_7α_ in the quail diencephalon.^75^ Luzindole, a melatonin receptor antagonist, abolishes the inhibitory action of melatonin on the biosynthesis of 7α‐OH pregnenolone.^75^ Accordingly, melatonin inhibits the biosynthesis of 7α‐OH pregnenolone in the quail diencephalon (for reviews, see 76, 77). Melatonin action on the biosynthesis of 7α‐OH pregnenolone was also found in the newt diencephalon (for a review, see 76). Based on these findings, it is considered that melatonin further regulates the biosynthesis of allopregnanolone not only in the brain but also in the pineal gland. Future studies focused on the interaction of pineal allopregnanolone and melatonin are needed to demonstrate the molecular mechanism of environmental light conditions affecting the survival of Purkinje cell during cerebellar development.

## 10 CONCLUSION AND FUTURE PROSPECTS

This review summarized new findings by the discovery of cerebellar and pineal allopregnanolone and their neuroprotective actions on the growth and survival of Purkinje cells during development.

**Figure 5:**
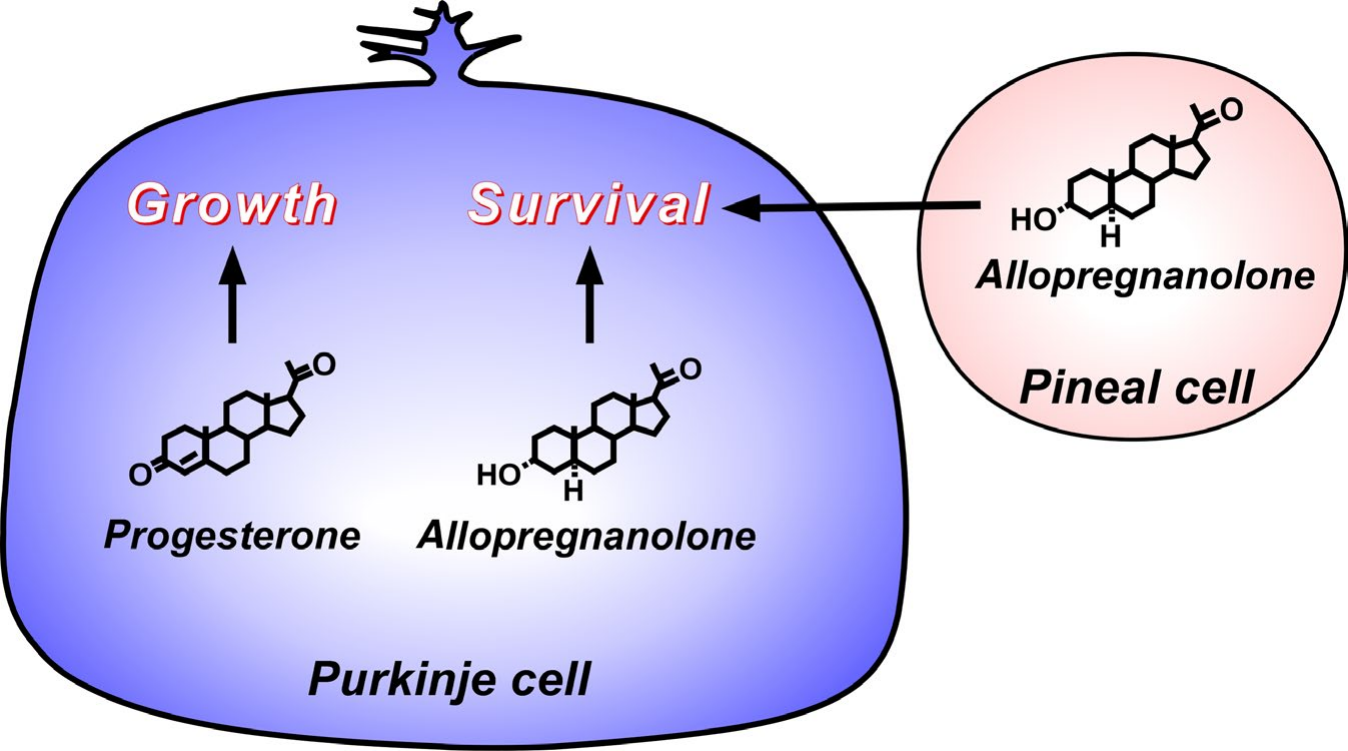
Summary of neurotrophic and neuroprotective actions of cerebellar and pineal neurosteroids on the growth and survival of Purkinje cells during cerebellar development. Progesterone produced in the Purkinje cell promotes dendritic growth, spinogenesis, and synaptogenesis *via* its cognate nuclear receptor by inducing the expression of BDNF, a neurotrophic factor, in the developing Purkinje cell. These neurotrophic actions of progesterone and estradiol contribute to the formation of the cerebellar neuronal circuit during cerebellar development. Moreover, allopregnanolone produced in the Purkinje cell and the pineal gland prevents the death of Purkinje cells in the cerebellum by suppressing the activity of caspase‐3, a crucial mediator of apoptosis. These neuroprotective actions of allopregnanolone may also contribute to the formation of the cerebellar neuronal circuit during the cerebellar development

The Purkinje cell, an important cerebellar neuron, was discovered as a major site for the biosynthesis of neurosteroids in the brain. This neuron actively synthesizes progesterone and allopregnanolone, a progesterone metabolite, de novo from cholesterol during cerebellar development when the cerebellar neuronal circuit formation occurs (Figures 1 and 5). Progesterone produced in the Purkinje cell promotes the dendritic growth, spinogenesis, and synaptogenesis of the Purkinje cell during cerebellar development (Figure 3). Furthermore, allopregnanolone produced in the Purkinje cell is involved in the survival of this neuron (Figure 3). This neuroprotective action of allopregnanolone may also contribute to the formation of the cerebellar neuronal circuit during cerebellar development by the mechanisms underlying the “intracrine” and/or “autocrine” actions of allopregnanolone in cerebellar Purkinje cells (Figure 3).

Subsequently, the pineal gland was discovered as a major site for the biosynthesis of neurosteroids. The pineal gland actively produces neurosteroids de novo from cholesterol (Figure 2). For the past 30 years, we believed that neurosteroids are produced only in neurons and glial cells in the brain and peripheral nervous system. This is a new concept of the biosynthesis of neurosteroids. Allopregnanolone, a major neurosteroid produced in the pineal gland, acts on the cerebellum to prevent the death of Purkinje cells in the cerebellum by suppressing the activity of caspase‐3, a crucial mediator of apoptosis, during cerebellar development (Figures 3 and 4). Light‐at‐night‐induced circadian disruption leads to cerebellar Purkinje cell death through pineal allopregnanolone‐dependent mechanisms during early post‐hatch life (Figure 4). Thus, the results suggest that modern nighttime artificial light exposure affects the development of the brain.

It is important to clarify the interaction of cerebellar and pineal allopregnanolone in the regulation of neuronal survival in the cerebellum during development (Figure 5). Future studies are also needed to investigate the unknown interactions of other neurosteroids produced in the cerebellum and pineal gland.

## CONFLICT OF INTEREST

The authors have no financial conflicts of interest.
